# Influence of Genetic Variants in EGF and Other Genes on Hematological Traits in Korean Populations by a Genome-Wide Approach

**DOI:** 10.1155/2015/914965

**Published:** 2015-04-30

**Authors:** Yun Kyoung Kim, Ji Hee Oh, Young Jin Kim, Mi Yeong Hwang, Sanghoon Moon, Siew-Kee Low, Atsushi Takahashi, Koichi Matsuda, Michiaki Kubo, Juyoung Lee, Bong-Jo Kim

**Affiliations:** ^1^Division of Structural and Functional Genomics, Center for Genome Science, National Institute of Health, Centers for Disease Control and Prevention, Chungcheongbuk-do 361-951, Republic of Korea; ^2^Center for Integrative Medical Sciences, RIKEN, Kanagawa, Japan; ^3^Institute of Medical Science, The University of Tokyo, Tokyo, Japan

## Abstract

Hematological traits are important health indicators and are used as diagnostic clinical parameters for human disorders. Recently, genome-wide association studies (GWAS) identified many genetic loci associated with hematological traits in diverse ethnic groups. However, additional GWAS are necessary to elucidate the breadth of genetic variation and the underlying genetic architecture represented by hematological metrics. To identify additional genetic loci influencing hematological traits (such as hematocrit, hemoglobin concentration, white blood cell count, red blood cell count, and platelet count), we conducted GWAS and meta-analyses on data from 12,509 Korean individuals grouped into population-based cohorts. Of interest is EGF, a factor plays a role in the proliferation and differentiation of hematopoietic progenitor cells. We identified a novel EGF variant, which associated with platelet count in our study (*P*_combined_ = 2.44 × 10^−15^). Our study also replicated 16 genetic associations related to five hematological traits with genome-wide significance (*P* < 5 × 10^−8^) that were previously established in other ethnic groups. Of these, variants influencing platelet count are distributed across several genes and have pleiotropic effects in coronary artery disease and dyslipidemia. Our findings may aid in elucidating molecular mechanisms underlying not only hematopoiesis but also inflammatory and cardiovascular diseases.

## 1. Introduction

Hematological metrics are used as essential medical indicators [1]. Maintenance of homeostasis is linked to physiological pathways that can be tested via blood chemistry panels [2]. Variation in hematological traits is heritable [3, 4]. Recently, genome-wide association studies (GWAS) have revealed hundreds of genetic loci associated with hematological traits [5–8]. Many of associated loci with hematological traits are shared between different ethnic groups. Despite success of discovery of large number of disease-associated variants, less than 10% of the heritability was explained by identified variants [9].

In addition, previous studies illustrated that significant differences in hematological traits exist between ethnic groups. For example, African Americans tend to have lower white blood cell counts, whereas persons of Japanese descent generally have fewer red blood cell-related anomalies than typically seen in other populations [10, 11]. These observations may suggest that there is a genetic basis for many hematological traits and investigation of unveiled variants is still required [1]. And also, previously identified common loci have yet to be thoroughly evaluated through a genome-wide scan in persons of Korean descent.

In this study, we sought to identify additional ethnic Korean-specific genetic variants associated with five hematological traits: hemoglobin (Hb), hematocrit (Hct), red blood cell count (RBC), white blood cell count (WBC), and platelet count (PLT). To achieve our aim, we thus carried out a GWAS and meta-analysis in Korean populations to look specifically for effects related to these metrics. Subsequently, we performed pleiotropic association analyses and functional annotation of the identified trait-associated loci. Our results may not only highlight the biologically important role of genetic variants in hematological traits found in Korean populations but also provide useful insight on understanding genetic diversity between ethnic groups.

## 2. Materials and Methods

### 2.1. Study Subjects

We performed GWAS based on 5 hematological traits (Hb, Hct, WBC, RBC, and PLT) with data from 12,509 subjects from two population-based cohorts that are comprised in the Korean Genome Epidemiology Study (KoGES). In discovery stage, we analyzed data for 8,842 subjects from the Korea Association Resource (KARE) project of KoGES [12]. To validate our discovery stage results, 3,667 healthy subjects in the Cardio Vascular Disease Association Study (CAVAS) of KoGES were used for the replication stage. For further replication of a novel locus, 8,053 subjects taking part in the Health2 study of KoGES and 23,032 Japanese subjects from the BioBank Japan project were selected for analyses. The descriptive statistics of each cohort are described in Supplementary Table 1 (available online at http://dx.doi.org/10.1155/2015/914965). And more detailed explanations of each cohort were previously described [6, 12, 13].

This study was approved by the ethics committee of the Korea Centers for Disease Control and Prevention's Institutional Review Board, and all of study subjects provided written informed consent prior to taking part in the study.

### 2.2. Phenotype Determination

Hematological trait values were available for up to 20,562 subjects (8,842 KARE subjects, 3,667 CAVAS subjects, and 8,053 Health2 subjects) taking part in KoGES. Fasting blood samples were drawn from study subjects into a test tube containing an anticoagulant (e.g., EDTA), and relevant traits were measured or calculated using an automated electronic cell counter, ADIVA 120 hematology system by Bayer Diagnostics, USA.

### 2.3. Genotyping and Quality Control

In the discovery stage, 10,004 KARE study samples were genotyped by the Affymetrix Genome-Wide Human SNP array 5.0. Our quality control criteria are as follows: samples (i) with missing genotype call rate (>4%), (ii) with excessive heterozygosity (>30%), (iii) with gender inconsistencies, and (iv) from subject with cancer; SNPs with (i) missing genotype call rate (>5%), (ii) low MAF (<0.01), and (iii) Hardy-Weinberg equilibrium (*P* < 1 × 10^−6^) were excluded. Following quality control analyses, data for 8,842 subjects and 352,228 SNPs were retained for further study. For* in silico* replication data, the 4,034 CAVAS samples were genotyped using the Illumina HumanOmni1-Quad BeadChip. The report file containing input signal intensity of samples was converted using the Illumina BeadStudio software package. Following quality control, 3,667 samples and 730,073 SNPs were deemed appropriate for analyses. Detailed quality control sample criteria and the genotypes from the two cohorts were described previously [12, 13].

For further replication of a novel locus, analyses in two methods were performed,* de novo* and* in silico* replication analysis. In* de novo* replication, we genotyped a SNP with the GoldenGate assay (Illumina Inc.) using 8,053 samples from Health2 study. The genotype success rate was 99.9%. In* in silico* replication, we used imputed data of 4q25 region based on genotype data using Illumina Human610-Quad BeadChip in 23,032 samples from the BioBank Japan project at the Center for Integrative Medical Sciences, RIKEN. The quality control criteria of Japanese samples and SNPs were described previously [6].

SNPs were imputed based on HapMap (phase 2, release 22, NCBI build 36 and dbSNP build 126; http://hapmap.ncbi.nlm.nih.gov/) data from the Japanese population in Tokyo, Japan (JPT), and the Han Chinese population in Beijing, China (CHB), using the IMPUTE program (version 2) (https://mathgen.stats.ox.ac.uk/impute/impute_v2.html) [14]. Following our quality control testing, 1,590,162 SNPs from the KARE study and 2,150,086 SNPs from the CAVAS study were used for further analyses [13, 15].

### 2.4. Statistical Analyses

To investigate the genetic causes for the five specified hematological traits, we carried out GWAS using a linear regression model via the PLINK program (http://pngu.mgh.harvard.edu/~purcell/plink/) [16]. Phenotypes used in the analyses were approximately normally distributed, and age and gender were incorporated into the analyses as covariates. We conducted meta-analyses for selected SNPs that exceeded our criteria of *P* < 1 × 10^−5^ in the discovery stage and *P* < 0.05 in the replication stage, with the inverse variance method using the METAL program (http://genome.sph.umich.edu/wiki/METAL) [17]. After meta-analyses, SNPs with the accepted genome-wide significance level (*P* < 5 × 10^−8^), which reflected testing of one million SNPs [18], were considered statistically significant.

### 2.5. Association Analyses with Related Traits (Coronary Artery Disease and Lipid Profiles)

As we were interested in the effects of genome-wide significant SNPs on PLT, associations of each SNP with the lipid profile metrics (total cholesterol (TC), triglyceride (TG), LDL-cholesterol (LDL), and HDL-cholesterol (HDL)) and CAD were implemented using 8,842 KARE subjects and CAD 2,123 cases and 2,690 controls that were previously published, respectively [15, 19]. Age and gender were used as covariates in all analyses.

## 3. Results

We conducted GWAS on 1,590,162 common SNPs (minor allele frequency (MAF) > 1%) and five hematological traits, namely, Hb, Hct, WBC, RBC, and PLT, for 8,842 subjects of the KARE project [12]. We carried forward SNPs of our top association results that satisfied the threshold (*P* < 1 × 10^−5^) for replication for 3,667 subjects in the CAVAS study, which represented a rural population-based cohort. Thirty-two variants were validated with statistical significance (*P* < 0.05) in CAVAS study (Supplementary Table 3). Descriptive information for the study samples and the inflation of test statistics (genomic control) are shown in Supplementary Table 1 and Supplementary Table 2, respectively. The quantile-quantile plots for five hematological traits are presented in Supplementary Figure 1.

**Figure 1:**
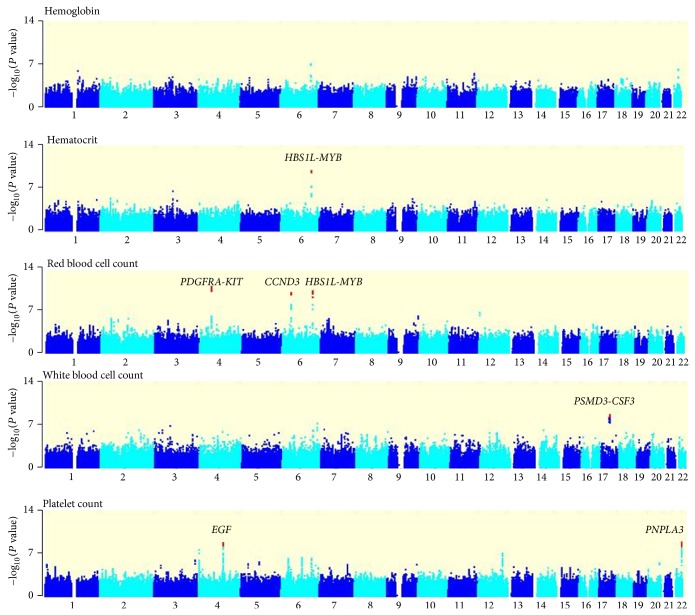
Manhattan plots of the GWAS for five hematological traits in discovery stage. Vertical axis indicates −log_10_ *P* values of SNPs in the GWAS for Hb, Hct, RBC, WBC, and PLT, and horizontal axis represents chromosomal position. The genetic loci that exceeded the genome-wide significance threshold of *P* < 5.0 × 10^−8^ are marked in red in each of the traits.

For the 12,509 data we used, we identified 17 genetic regions including one novel genetic association for PLT (4q25, on the EGF gene) that reached our threshold for genome-wide significance (*P* < 5 × 10^−8^), one for Hb, six for RBC, two for WBC, and six for PLT and one region (6q23.3) associated with three traits (Hct, RBC, and PLT) (Table 1 and Figure 1).

### 3.1. Previously Reported Loci

Of the 17 regions we identified, seven included previously reported associations of erythrocyte-related traits (Hb, Hct, and RBC) with the following loci: 22q12.3 (TMPRSS6), 6q23.3 (HBS1L-MYB), 4q12 (PDGFRA-KIT), 6p21.1 (CCND3), 12p13.3 (PARP11-CCND2), 9q34.2 (ABO), and 2p21 (PRKCE) (Table 1) [6]. Our analyses of WBC also revealed two previously reported loci with rs8070454 located in PSMD3-CSF3 (17q21.1) and rs11981340 in CDK6 (7q21.2) (Table 1) [6]. GWAS for PLT showed previously reported associations with the following six loci: 22q13.31 (PNPLA3), 6p21.33 (LY6G5C), 6p21.32 (HLA-DOA-HLA-DPA1), 12q24.12 (SH2B3), 6q23.3 (HBS1L-MYB), and 3q27.1 (THPO) (Table 1) [6, 20]. Other known loci were also scanned using the GWAS catalog of the National Human Genome Research Institute (Supplementary Table 4) [21].

### 3.2. A Newly Identified Locus

**Figure 2:**
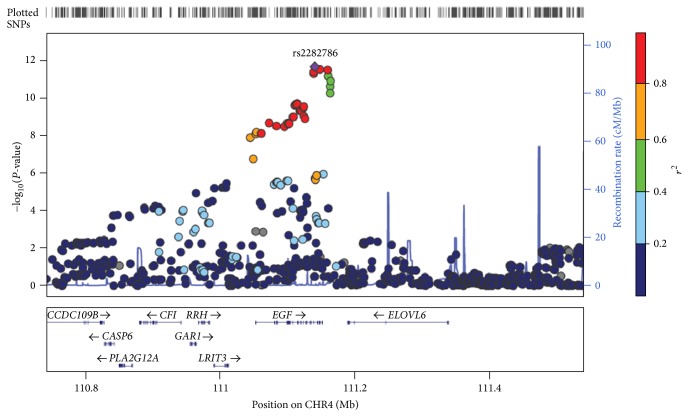
A regional association plot of the novel genetic locus associated with PLT. Round shaped dots represent −log_10_ *P* values of SNPs from combined meta-analyses (discovery and replication stages). Diamond shaped purple dot that is located in the center of 0.8 Mb of 4q25 genomic region indicates the strongest signal associated with PLT. The color of each dot indicates the level of linkage disequilibrium (LD), *r* ^2^, relative to the SNP rs2282786. At the bottom, the locations of RefSeq genes are represented. Plot was generated from JPT + CHB panel based on hg18 HapMap phase 2 using LocusZoom.

We identified a novel intronic variant, rs2282786, located on EGF at 4q25 that associated with lower platelet counts (effect size = −6.64 ± 0.944, *P* = 2.05 × 10^−12^) (Table 1 and Figure 2). This variant also showed statistical significance in subsequent replication stages that consisted of two populations, including 8,053 subjects from the Health2 cohort [12] and 23,032 subjects from Japanese population [6] (*P*_combined_ = 2.44 × 10^−15^) (Table 3). We also found a monomorphic ethnic difference in allele frequency of rs2282786 (T allele) in those of European and Yoruba descent in contrast to those of Asian descent (CHB + JPT, MAF = 0.258) based on HapMap project data (http://hapmap.ncbi.nlm.nih.gov/) (Supplementary Figure 2).

### 3.3. Pleiotropic Effect of PLT Related Variants on CAD and Lipid Profile

We examined associations between seven PLT-associated variants with genome-wide significance and other traits related to CAD and lipid profile, including TC, TG, LDL, and HDL (Table 2). Two variants near HBS1L-MYB and PNPLA3 were associated with three lipids (TC, TG, and LDL), respectively. Rs739496, located on 3′-UTR of SH2B3, was associated with both decreased platelet count and a decreased risk for CAD (Table 2). Other variants did not have compelling associations with these five traits.

## 4. Discussion

Recently, numerous genetic loci for hematological traits were discovered through several GWASs of European, African American, and Japanese populations [5–7, 22]. Using a similar approach, we screened data for 12,509 Korean individuals and confirmed the participation of 16 known loci associated with hematological traits and also identified one novel genetic locus affecting PLT. The SNP rs2282786 located on EGF in 4q25 showed a strong association with PLT with genome-wide significance (*P*_combined_ = 2.44 × 10^−15^) by combined meta-analysis in 43,594 individuals (20,562 Koreans and 23,032 Japanese) (Table 1, Figure 2, and Table 3). This SNP also showed an ethnicity-based difference in allele frequency (Supplementary Figure 2). This is a compelling discovery and provides evidence of a divergent genetic background based on ethnic differences seen in hematological traits.

Additionally, to examine the association between genetic variants and the level of gene expression, the novel PLT-associated locus was cross-referenced with expression quantitative trait loci (eQTL) associations using genetic variation and gene expression profiling data from Gene Expression Variation (GENEVAR) (http://www.sanger.ac.uk/resources/software/genevar) [23]. These data were based on lymphoblastoid cell lines (LCLs) from 162 HapMap3 individuals (80 CHB + 82 JPT) [24]. An intronic SNP, rs4698756 on EGF, in weak linkage disequilibrium (LD) (*r* ^2^ = 0.229, *D*′ = 0.787) with rs2282786, showed a statistical significant* cis*-regulatory effect on gene expression levels of EGF in Chinese populations (*P* = 0.0401) (data are not shown). Furthermore, to elucidate the regulatory function of the locus, we surveyed the Encyclopedia of DNA Elements (ENCODE) features such as regulatory chromatin states, DNAse hypersensitivity, and ChIP-seq experiment using UCSC Genome Browser (http://genome.ucsc.edu/). According to the functional annotation based on ENCODE data, rs4698756 lies within regulatory functional elements comprising transcription factor binding sites, DNase clusters, and proteins required for chemical modification of histones. Even though the extent of LD between rs2282786 and rs4698756 was not so strong to use rs4698756 as a direct surrogate of rs2282786, this functional information may suggest the possibility of the regulation of EGF expression that may modulate platelet counts.

The EGF gene encodes epidermal growth factor; the encoded protein acts as a potent mitogenic factor, playing an important role in the growth, proliferation, and differentiation of numerous cell types [MIM: 131530]. It may play a role in growth, proliferation, and differentiation of megakaryocytes and platelet production. Previous studies reported that activated platelets induced by inflammation may secrete EGF and proinflammatory substances for subsequent thrombus formation in an inflammation-hemostasis cycle that is a tightly interrelated pathophysiologic process [25, 26]. It is well known that platelets play an important role in CAD both in the pathogenesis of atherosclerosis and in the development of acute thrombotic events. Accordingly, high blood lipid levels can enhance platelet aggregation, causing CAD [27]. The resulting associations related to the identified PLT, CAD, and lipid profile loci suggested that they also have pleiotropic effects in the process of CAD and dyslipidemia (Table 2). Two variants located on HBS1L-MYB and PNPLA3, and one variant on SH2B3, that associated with PLT, also were significantly associated with lipids (TC, TG, and LDL) and CAD, respectively. Among them, SH2B3 encodes a member of the SH2B adaptor family of proteins [MIM: 605093] that plays a critical role in hematopoiesis involving blood coagulation and erythropoietin signaling pathways [28]. This gene has previously been found to be associated with type 1 diabetes [29], cardiovascular diseases [30], and hypertension [31].

To date, many studies have reported genetic factors associated with hematological traits via GWAS across diverse ethnic groups [5–7, 22]. According to previous transethnic studies, the most commonly revealed variants were replicated in all of ethnic groups and have a species-wide role in biological pathways of hematopoiesis. However, these common genetic loci may account for a low percentage of hematological trait heritability [32]. Therefore, to identify genetic mechanisms underlying these traits, additional large-scale population based studies incorporating multiple rare variants or gene-gene and gene-environment interactions should be undertaken. And performing functional experiments may also help to validate the results of statistical analyses for human hematological traits.

In summary, we illustrated that a genome-wide approach identified genetic variants contributing to phenotypic variation of hematological traits in Korean populations. We identified one novel ethnic specific variant associated with PLT that localized to a key regulator of hematopoiesis and confirmed previously implicated loci that were associated five hematological traits. We also provided pleiotropic effects of PLT-associated variants that may support the biological role of genetic determinants for hematological traits. Our findings may help identify biological pathways that contribute not only to hematopoiesis but also to inflammatory and cardiovascular diseases in humans.

## Supplementary Material

Supplementary Figure 1: The quantile - quantile plots for five hematological traits in discovery stage.
Supplementary Figure 2: Population diversity of rs2282786.
Supplementary Table 1: Descriptive statistics of samples analyzed in this study.
Supplementary Table 2: Descriptive statistics of samples analyzed in this study.
Supplementary Table 3: Association results of 32 variants that were verified in the replication stage.
Supplementary Table 4: Replication of previously reported loci associated with hematological traits in Korean populations.

## Figures and Tables

**Table 1:** Results of genome-wide association analyses for hematological traits.

Trait	CHR	SNP	Cytoband	Gene	Minor allele	Discovery (*n* = 8,842)			Replication (*n* = 3,667)		Combined (*n* = 12,509)		
						MAF	Effect size	*P*	Effect size	*P*	Effect size	*P*	*P*_het_ (*Q*)
Novel													
PLT	4	rs2282786	4q25	EGF	C	0.25	−6.66 ± 1.123	3.18 × 10^−9^	−6.58 ± 1.741	1.58 × 10^−4^	−6.64 ± 0.944	2.05 × 10^−12^	0.97 (0.00)
Previously reported													
Hb	22	rs2076086	22q12.3	TMPRSS6	T	0.49	0.09 ± 0.018	7.33 × 10^−7^	0.11 ± 0.026	3.24 × 10^−5^	0.09 ± 0.015	1.19 × 10^−10^	0.52 (0.41)
Hct	6	rs9376090	6q23.3	HBS1L-MYB	C	0.32	−0.35 ± 0.055	1.68 × 10^−10^	−0.18 ± 0.081	2.68 × 10^−2^	−0.30 ± 0.045	6.20 × 10^−11^	0.08 (3.05)
RBC	6	rs7775698	6q23.3	HBS1L-MYB	T	0.33	−0.07 ± 0.006	6.82 × 10^−34^	−0.06 ± 0.009	4.92 × 10^−12^	−0.07 ± 0.005	2.69 × 10^−44^	0.20 (1.68)
	4	rs17084406	4q12	PDGFRA-KIT	G	0.26	−0.04 ± 0.006	2.09 × 10^−11^	−0.05 ± 0.010	1.81 × 10^−8^	−0.05 ± 0.005	2.85 × 10^−18^	0.36 (0.85)
	6	rs3218108	6p21.1	CCND3	T	0.22	0.04 ± 0.007	2.15 × 10^−10^	0.05 ± 0.010	2.54 × 10^−6^	0.04 ± 0.006	2.59 × 10^−15^	0.79 (0.07)
	12	rs7138216	12p13.3	PARP11-CCND2	C	0.27	−0.03 ± 0.006	2.70 × 10^−7^	−0.03 ± 0.009	4.73 × 10^−3^	−0.03 ± 0.005	5.19 × 10^−9^	0.54 (0.37)
	9	rs8176743	9q34.2	ABO	T	0.21	0.03 ± 0.007	9.88 × 10^−7^	0.03 ± 0.010	5.73 × 10^−3^	0.03 ± 0.006	2.12 × 10^−8^	0.62 (0.25)
	2	rs2218660	2p21	PRKCE	G	0.18	−0.03 ± 0.007	2.90 × 10^−6^	−0.03 ± 0.011	2.90 × 10^−3^	−0.03 ± 0.006	2.94 × 10^−8^	0.85 (0.04)
WBC	17	rs8070454	17q21.1	PSMD3-CSF3	T	0.47	0.16 ± 0.028	3.62 × 10^−9^	0.11 ± 0.043	8.68 × 10^−3^	0.15 ± 0.023	1.74 × 10^−10^	0.31 (1.04)
	7	rs11981340	7q21.2	CDK6	C	0.34	−0.13 ± 0.029	4.99 × 10^−6^	−0.20 ± 0.045	9.58 × 10^−6^	−0.15 ± 0.024	4.33 × 10^−10^	0.21 (1.58)
PLT	22	rs1977081	22q13.31	PNPLA3	C	0.41	−5.84 ± 0.997	4.70 × 10^−9^	−3.76 ± 1.608	1.94 × 10^−2^	−5.27 ± 0.847	5.10 × 10^−10^	0.27 (1.21)
	6	rs9469032	6p21.33	LY6G5C	G	0.03	12.7 ± 2.757	4.42 × 10^−6^	16.6 ± 4.336	1.35 × 10^−4^	13.8 ± 2.327	3.12 × 10^−9^	0.45 (0.58)
	6	rs9277053	6p21.32	HLA-DOA-HLA-DPA1	A	0.34	5.18 ± 1.049	7.95 × 10^−7^	5.27 ± 1.622	1.18 × 10^−3^	5.21 ± 0.881	3.43 × 10^−9^	0.96 (0.00)
	12	rs739496	12q24.12	SH2B3	A	0.11	−8.36 ± 1.572	1.06 × 10^−7^	−5.75 ± 2.385	1.06 × 10^−2^	−7.57 ± 1.313	8.00 × 10^−9^	0.36 (0.84)
	6	rs9399137	6q23.3	HBS1L-MYB	C	0.33	5.24 ± 1.072	1.06 × 10^−6^	5.00 ± 1.630	2.20 × 10^−3^	5.17 ± 0.896	8.07 × 10^−9^	0.90 (0.02)
	3	rs13091574	3q27.1	THPO	C	0.16	6.39 ± 1.319	1.28 × 10^−6^	6.22 ± 2.142	3.73 × 10^−3^	6.34 ± 1.123	1.62 × 10^−8^	0.94 (0.01)

CHR, chromosome; BP, base position; MAF, minor allele frequency; Hb, hemoglobin; Hct, hematocrit; RBC, red blood cell count; WBC, white blood cell count; PLT, platelet count. Effect sizes are shown as beta ± S.E. A test of heterogeneity (*P*_het_) was conducted; *Q*, Cochrane's *Q* value based on chi-squared statistics. Age and gender were used in analyses as covariates.

**Table 2:** Associations with CAD and lipid profiles for loci-associated PLT with genome-wide significance.

Trait	SNP	Cytoband	Gene	CAD (*n* = 4,813)		TC (*n* = 8,842)		TG (*n* = 8,842)		LDL (*n* = 8,842)		HDL (*n* = 8,842)	
				OR (95% CI)	*P*	Effect size	*P*	Effect size	*P*	Effect size	*P*	Effect size	*P*
PLT	rs13091574	3q27.1	THPO	0.96 (0.86–1.07)	0.4571	−0.39 ± 0.72	0.5944	−1.63 ± 2.12	0.4426	−0.67 ± 0.65	0.3096	0.37 ± 0.21	0.0704
	rs2282786	4q25	EGF	1.07 (0.97–1.17)	0.1803	−0.33 ± 0.62	0.5954	−2.40 ± 1.82	0.188	−0.05 ± 0.56	0.9282	−0.05 ± 0.17	0.7807
	rs9469032	6p21.33	LY6G5C	1.15 (0.93–1.42)	0.1943	1.69 ± 1.51	0.2613	4.37 ± 4.47	0.3288	1.00 ± 1.37	0.4655	0.01 ± 0.43	0.9818
	rs9277053	6p21.32	HLA-DOA-HLA-DPA1	—	—	0.93 ± 0.57	0.1059	0.16 ± 1.67	0.9252	0.79 ± 0.52	0.1296	0.14 ± 0.16	0.3875
	rs9399137	6q23.3	HBS1L-MYB	—	—	−2.79 ± 0.58	1.87 × 10^−6^	−5.75 ± 1.72	8.41 × 10^−4^	−1.78 ± 0.53	8.02 × 10^−4^	−0.05 ± 0.17	0.7750
	rs739496	12q24.12	SH2B3	0.82 (0.72–0.94)	3.70 × 10^−3^	0.99 ± 0.86	0.2522	4.77 ± 2.54	0.06059	0.05 ± 0.78	0.9487	0.36 ± 0.24	0.1420
	rs1977081	22q13.31	PNPLA3	0.94 (0.87–1.03)	0.182	−2.03 ± 0.55	2.08 × 10^−4^	−5.94 ± 1.60	2.00 × 10^−4^	−1.30 ± 0.50	8.93 × 10^−3^	0.07 ± 0.16	0.6558

CHR, chromosome; BP, base position; PLT, platelet count; CAD, coronary artery disease; TC, total cholesterol; TG, triglyceride; LDL, LDL-cholesterol; HDL, HDL-cholesterol. Logistic and linear regression analyses adjusted by age and gender were performed. Effect sizes are shown as beta ± S.E.

**Table 3:** Association results of rs2282786 for platelet count.

Trait	CHR	SNP	BP	Gene	Function	MA	KARE + CAVAS (*n* = 12,509)		Health2^∗^ (*n* = 8,053)		BioBank Japan (*n* = 23,032)		Combined (*n* = 43,594)		
							Effect size	*P*	Effect size	*P*	Effect size	*P*	Effect size	*P*	*P*_het_ (*Q*)
PLT	4	rs2282786	111141945	EGF	Intronic	C	−6.64 ± 0.944	2.05 × 10^−12^	−4.21 ± 1.11	1.59 × 10^−4^	−4.78 ± 1.04	4.47 × 10^−6^	−5.23 ± 0.66	2.44 × 10^−15^	0.41 (0.68)

^∗^The association result of rs11098063, a proxy SNP of rs2282786 (*r* ^2^ > 0.9), in Health2 cohort is represented in this table. Effect sizes are shown as beta ± S.E.; CHR, chromosome; BP, base position; MA, minor allele.
