# Supplementary material for: Influence of Genetic Variants in EGF and Other Genes on Hematological Traits in Korean Populations by a Genome-Wide Approach
Source: Biomed Res Int. 2015 Apr 30;2015:914965. doi: 10.1155/2015/914965 (PMC4430676; doi:10.1155/2015/914965)
Supplement: Supplementary file 1 — Supplementary Figure 1: The quantile - quantile plots for five hematological traits in discovery stage. Supplementary Figure 2: Population diversity of rs2282786. Supplementary Table 1: Descriptive statistics of samples analyzed in this study. Supplementary Table 2: Descriptive statistics of samples analyzed in this study. Supplementary Table 3: Association results of 32 variants that were verified in the replication stage. Supplementary Table 4: Replication of previously reported loci associated with hematological traits in Korean populations. [file 914965.f1.pdf]

Supplementary Information

Influence of genetic variants in EGF and other genes on hematological traits in Korean populations by a genome-wide approach

Yun Kyoung Kim[1]*, Ji Hee Oh[1]*, Young Jin Kim[1], Mi Yeong Hwang[1], Sanghoon Moon[1], Siew-Kee Low[2], Atsushi Takahashi[2], Koichi Matsuda[3], Michiaki Kubo[2], Juyoung Lee[1] and Bong-Jo Kim[1§]

[1]Division of Structural and Functional Genomics, Center for Genome Science, National Institute of Health, Centers for Disease Control and Prevention, Chungcheongbuk-do, Korea, [2]Center for Integrative Medical Sciences, RIKEN, Kanagawa, Japan, [3]Institute of Medical Science, The University of Tokyo, Tokyo, Japan

*These authors contributed equally to this work.

[§]Corresponding author:

Bong-Jo Kim, Ph.D.

Division of Structural and Functional Genomics, Center for Genome Science, National Institute of Health, Centers for Disease Control and Prevention, Chungcheongbuk-do, Korea, 361-951, Phone : +82-43-719-8870, FAX : +82-43-719-8908, E-mail : kbj6181@cdc.go.kr

Supplementary Figure 1 The quantile - quantile plots for five hematological traits in discovery stage showed genome-wide distribution of the empirically observed of test statistic in comparison with the expected null distribution. (a) is plots for Hb, (b) is for Hct, (c) is for RBC, (d) is for WBC and (e) is for PLT.

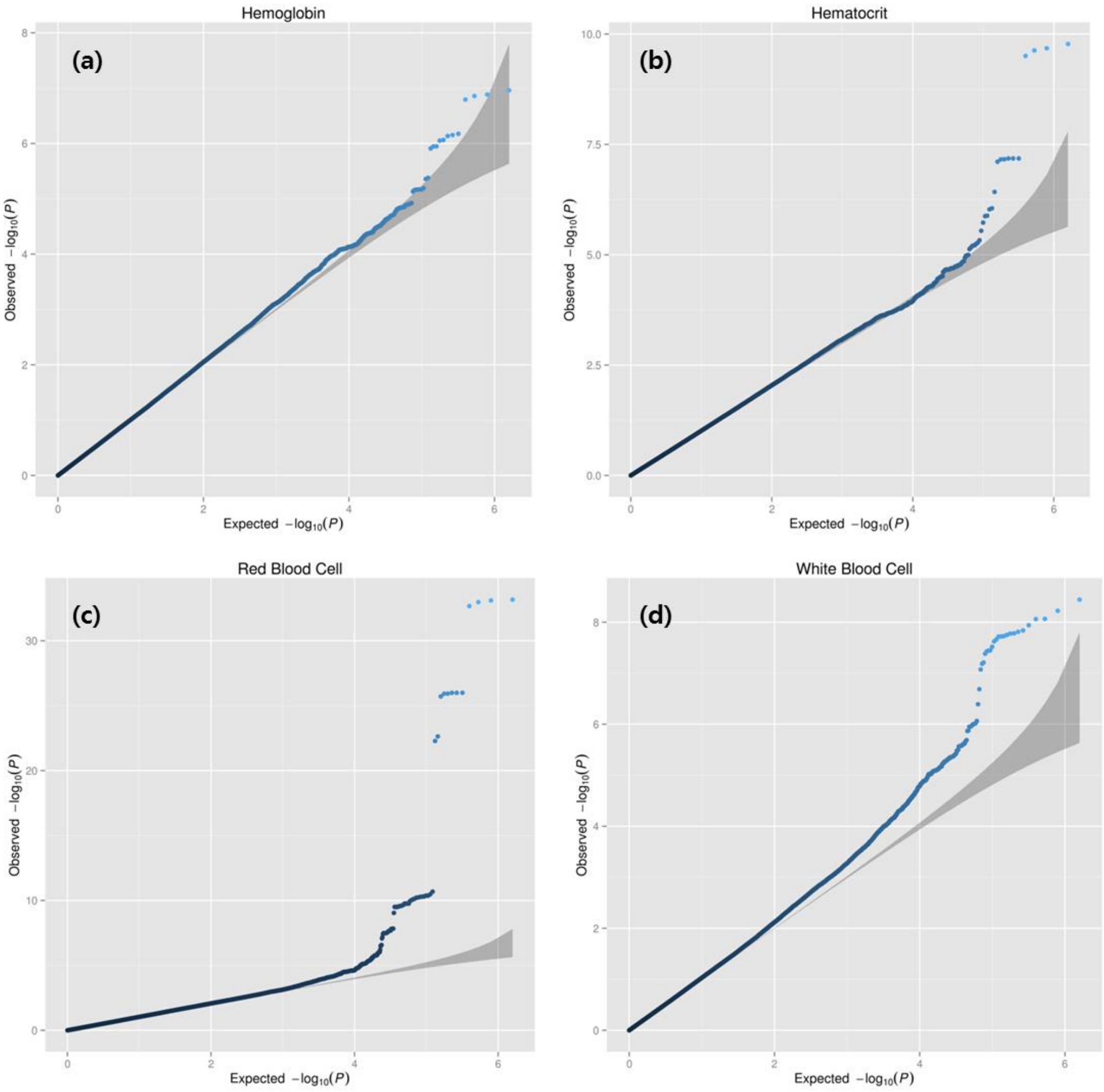

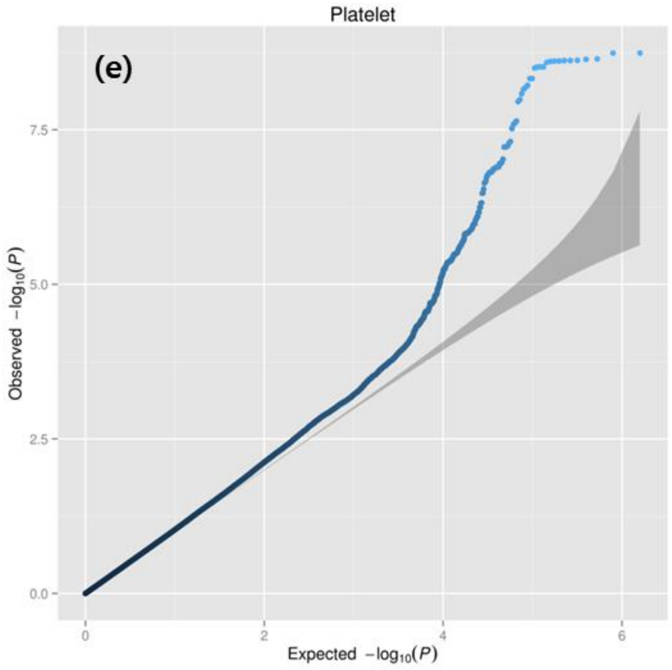
Platelet
(e)
Observed −log10(P)
Expected −log10(P)
0.0
2.5
5.0
7.5
0
2
4
6

Supplementary Figure 2 Population diversity of rs2282786 referring from Database of Single Nucleotide Polymorphism (dbSNP, http://www.ncbi.nlm.nih.gov/snp/)

Sample Ascertainment				Genotype Detail				Alleles	
Population	Individual Group	Chrom. Sample Cnt.	Source	C/C	C/T	T/T	HWP	C	T
AFD_EUR_PANEL	European	48	IG			1.000			1.000
AFD_AFR_PANEL	African American	46	IG			1.000			1.000
AFD_CHN_PANEL	Asian	46	IG	0.087	0.652	0.261	0.100	0.413	0.587
HapMap-CEU	European	120	IG			1.000			1.000
HapMap-HCB	Asian	90	IG	0.089	0.356	0.556	0.584	0.267	0.733
HapMap-JPT	Asian	86	IG	0.070	0.326	0.605	0.584	0.233	0.767
HapMap-YRI	Sub-Saharan African	118	IG			1.000			1.000
pilot_1_CHB+JPT_low_coverage_panel		120	AF					0.258	0.742
JBIC-allele		728	AF					0.211	0.789

Supplementary Table 1. Descriptive statistics of samples analyzed in this study

Traits		Discovery		Replication 1		Replication 2		Replication 3	
		KARE (n=8,842)		CAVAS (n=3,667)		Health2 (n=8,053)		BioBank Japan (n=23,032)	
Age		52.2 ± 8.92		59.8 ± 10.05		56.8 ± 7.85		63.5 ± 11.94	
Sex	male (%)	4,183 (47.3%)		1,375 (37.5%)		3,281 (40.7%)		12,366 (53.7%)	
	female (%)	4,659 (52.7%)		2,292 (62.5%)		4,762 (59.1%)		10,666 (46.3%)	
		mean ± SD	CV(%)	mean ± SD	CV(%)	mean ± SD	CV(%)	mean ± SD	CV(%)
Hemoglobin (g/dL)		13.6 ± 1.60	11.8	13.6 ± 1.43	10.5	-	-	-	-
Hematocrit (%)		41.0 ± 4.60	11.2	40.4 ± 4.15	10.3	-	-	-	-
RBC (× $10^6/\mu$L)		4.42 ± 0.49	11.1	4.34 ± 0.42	9.7	-	-	-	-
WBC (× $10^3/\mu$L)		6.59 ± 1.84	27.9	6.57 ± 1.87	28.5	-	-	-	-
Platelet count (× $10^3/\mu$L)		266.3 ± 65.3	24.5	258.6 ± 62.0	24.0	258.0 ± 62.4	24.2	231.1 ± 90.9	39.3

n, sample size; RBC, red blood cell count; WBC, white blood cell count; CV, coefficient of variation;

KARE, Korean Association Resource Project; CAVAS, Cardiovascular disease Association Study;

Data are shown as mean ± standard deviation.

Supplementary Table 2. Genomic inflation factors of analyses in the discovery stage

Trait	Inflation factor
Hb	1.003623
Hct	1.016721
RBC	1.035321
WBC	1.029904
PLT	1.036225

Genomic inflation factor is defined as the ratio of the median of the observed distribution of the test statistic to the expected median.

Supplementary Table 3. Association results of 32 variants that were verified in the replication stage.

Trait	CHR	SNP	Cytoband	BP	Gene	A1/A2	MAF	Discovery (KARE, n=8,842)		Replication (CAVAS, n=3,667)		Combined (n=12,509)		
								effect size	*P*	effect size	*P*	effect size	*P*	P_{het}(Q)
Hb	22	rs2076086	22q12.3	35800482	TMPRSS6	T/C	0.49	0.09 ± 0.02	7.33E-07	0.11 ± 0.03	3.24E-05	0.09 ± 0.01	1.19E-10	0.52(0.41)
	2	rs2218660	2p21	46201296	PRKCE	G/T	0.18	-0.09 ± 0.02	6.51E-05	-0.09 ± 0.03	6.04E-03	-0.09 ± 0.02	1.25E-06	0.95(0.00)
	6	rs2523554	6p21.33	31439808	HLA-B-MICA	C/T	0.12	0.11 ± 0.03	9.34E-05	0.10 ± 0.04	1.44E-02	0.11 ± 0.02	4.06E-06	0.87(0.03)
Hct	6	rs9376090	6q23.3	135452921	HBS1L-MYB	C/T	0.32	-0.35 ± 0.05	1.68E-10	-0.18 ± 0.08	2.68E-02	-0.30 ± 0.05	6.20E-11	0.08(3.05)
	2	rs2218660	2p21	46201296	PRKCE	G/T	0.18	-0.30 ± 0.07	4.69E-06	-0.27 ± 0.10	5.84E-03	-0.29 ± 0.05	9.22E-08	0.81(0.06)
RBC	6	rs7775698	6q23.3	135460328	HBS1L-MYB	T/C	0.33	-0.07 ± 0.01	6.82E-34	-0.06 ± 0.01	4.92E-12	-0.07 ± 0.01	2.69E-44	0.20(1.68)
	4	rs17084406	4q12	55056941	PDGFRA-KIT	G/A	0.26	-0.04 ± 0.01	2.09E-11	-0.05 ± 0.01	1.81E-08	-0.05 ± 0.01	2.85E-18	0.36(0.85)
	6	rs3218108	6p21.1	42010633	CCND3	T/C	0.22	0.04 ± 0.01	2.15E-10	0.05 ± 0.01	2.54E-06	0.04 ± 0.01	2.59E-15	0.79(0.07)
	12	rs7138216	12p13.32	4204569	PARP11-CCND2	C/G	0.27	-0.03 ± 0.01	2.70E-07	-0.03 ± 0.01	4.73E-03	-0.03 ± 0.01	5.19E-09	0.54(0.37)
	9	rs8176743	9q34.2	135121236	ABO	T/C	0.21	0.03 ± 0.01	9.88E-07	0.03 ± 0.01	5.73E-03	0.03 ± 0.01	2.12E-08	0.62(0.25)
	2	rs2218660	2p21	46201296	PRKCE	G/T	0.18	-0.03 ± 0.01	2.90E-06	-0.03 ± 0.01	2.90E-03	-0.03 ± 0.01	2.94E-08	0.85(0.04)
	6	rs11153167	6q21	109729275	CCDC162P	T/A	0.11	-0.04 ± 0.01	5.10E-05	-0.03 ± 0.01	3.72E-02	-0.03 ± 0.01	6.12E-06	0.57(0.33)
	8	rs16887645	8q23.3	116767229	TRPS1-LINC00536	A/C	0.08	-0.04 ± 0.01	7.00E-05	-0.04 ± 0.02	2.13E-02	-0.04 ± 0.01	4.49E-06	0.75(0.10)
	16	rs11859733	16q21	56889274	PRSS54-GINS3	C/T	0.26	-0.03 ± 0.01	8.56E-05	-0.03 ± 0.01	3.62E-03	-0.03 ± 0.01	1.01E-06	0.96(0.00)
WBC	17	rs8070454	17q21.1	35414280	PSMD3-CSF3	T/C	0.47	0.16 ± 0.03	3.62E-09	0.11 ± 0.04	8.68E-03	0.15 ± 0.02	1.74E-10	0.31(1.04)
	3	rs10936603	3q26.2	171028346	LRRIQ4	G/T	0.32	-0.14 ± 0.03	4.42E-06	-0.09 ± 0.05	4.36E-02	-0.12 ± 0.02	7.40E-07	0.41(0.68)
	7	rs11981340	7q21.2	92153596	CDK6	C/T	0.34	-0.13 ± 0.03	4.99E-06	-0.20 ± 0.04	9.58E-06	-0.15 ± 0.02	4.33E-10	0.21(1.58)
	2	rs3729569	2q21.3	136319775	MCM6	T/G	0.18	0.15 ± 0.04	6.87E-05	0.16 ± 0.06	3.59E-03	0.15 ± 0.03	8.33E-07	0.79(0.07)
	4	rs9760319	4q28.3	138631979	NONE-PCDH18	G/A	0.43	0.11 ± 0.03	9.17E-05	0.09 ± 0.04	3.94E-02	0.10 ± 0.02	1.04E-05	0.73(0.12)
PLT	4	rs2282786	4q25	111141945	EGF	C/T	0.25	-6.66 ± 1.12	3.18E-09	-6.58 ± 1.74	1.58E-04	-6.64 ± 0.94	2.05E-12	0.97(0.00)
	22	rs1977081	22q13.31	42661461	PNPLA3	C/T	0.41	-5.84 ± 1.00	4.70E-09	-3.76 ± 1.61	1.94E-02	-5.27 ± 0.85	5.10E-10	0.27(1.21)
	12	rs739496	12q24.12	110372042	SH2B3	A/G	0.11	-8.36 ± 1.57	1.06E-07	-5.75 ± 2.39	1.60E-02	-7.57 ± 1.31	8.00E-09	0.36(0.84)

6	rs9277053	6p21.32	33114200	HLA-DOA-HLA-DPA1	A/G	0.34	5.18 ± 1.05	7.95E-07	5.27 ± 1.62	1.18E-03	5.21 ± 0.88	3.43E-09	0.96(0.00)
6	rs9399137	6q23.3	135460711	HBS1L-MYB	C/T	0.33	5.24 ± 1.07	1.06E-06	5.00 ± 1.63	2.20E-03	5.17 ± 0.90	8.07E-09	0.90(0.02)
3	rs13091574	3q27.1	185574799	THPO	C/A	0.16	6.39 ± 1.32	1.28E-06	6.22 ± 2.14	3.73E-03	6.34 ± 1.12	1.62E-08	0.94(0.01)
6	rs9469032	6p21.33	31753405	LY6G5C	G/C	0.03	12.7 ± 2.76	4.42E-06	16.6± 4.34	1.35E-04	13.8 ± 2.33	3.12E-09	0.45(0.58)
6	rs5745582	6p21.31	33654476	BAK1	T/C	0.22	5.03 ± 1.16	1.52E-05	5.39 ± 1.81	2.88E-03	5.14 ± 0.98	1.48E-07	0.87(0.03)
11	rs7946919	11q23.3	118646042	CBL	G/A	0.44	4.42 ± 1.03	1.83E-05	4.84 ± 1.56	1.92E-03	4.55 ± 0.86	1.23E-07	0.82(0.05)
13	rs4519241	13q31.1	84301372	MIR548F1-SLITRK6	C/A	0.40	-4.18 ± 1.01	3.26E-05	-3.82 ± 1.60	1.70E-02	-4.08 ± 0.85	1.67E-06	0.85(0.04)
22	rs2266950	22q11.21	19566372	SNAP29	A/G	0.05	9.57 ± 2.32	3.61E-05	7.14 ± 3.60	4.71E-02	8.86 ± 1.95	5.36E-06	0.57(0.32)
7	rs10260466	7p12.1	53688906	POM121L12-FLJ45974	A/C	0.22	-4.69 ± 1.18	7.11E-05	-4.36 ± 1.83	1.71E-02	-4.59 ± 0.99	3.60E-06	0.88(0.02)
17	rs6065	17p13.2	4777161	GP1BA	T/C	0.09	6.81 ± 1.74	8.82E-05	8.89 ± 2.58	5.78E-04	7.46 ± 1.44	2.25E-07	0.50(0.45)

A1 is defined as minor allele and A2 is major allele.

Supplementary Table 4. Replication of previously reported loci associated with hematological traits in Korean populations (SNP ID perfectly matched).

Trait	SNP	CHR	BP	Gene	A1/A2	Previously implicated				Replicated in Korean population (n=8,842)	
						MAF	effect size	*P*	Reference	effect size	*P*
Hb	rs10495928	2	46206670	PRKCE	G/A	0.34	0.06 ± 0.009	7.05×10^{-13}	[1]	-0.07 ± 0.023	1.17E-03
	rs495828	9	135144688	ABO	T/G	0.28	-0.09 ± 0.013	1.18×10^{-11}	[2]	-0.08 ± 0.020	2.26E-05
	rs6013509	20	50751758	TSHZ2	A/G	0.18	-0.06 ± 0.010	1.05×10^{-10}	[1]	-0.02 ± 0.019	0.2118
	rs5756504	22	35797216	TMPRSS6	T/C	0.45	0.08 ± 0.012	1.64×10^{-10}	[2]	0.09 ± 0.017	8.65E-07
Hct	rs10168349	2	46208555	PRKCE	C/G	0.34	0.19 ± 0.024	3.75×10^{-15}	[1]	-0.26 ± 0.065	6.26E-05
	rs7775698	6	135460328	HBS1L-MYB	T/C	0.35	-0.08 ± 0.012	9.52×10^{-11}	[2]	-0.35 ± 0.055	2.10E-10
	rs7385804	7	100073906	TFR2	C/A	0.38	-0.15 ± 0.024	4.45×10^{-10}	[1]	0.06 ± 0.086	0.5114
	rs495828	9	135144688	ABO	T/G	0.28	-0.08 ± 0.013	6.06×10^{-10}	[2]	-0.22 ± 0.057	9.26E-05
	rs2413450	22	35800170	TMPRSS6	T/C	0.38	-0.17 ± 0.024	1.85×10^{-13}	[1]	0.19 ± 0.050	1.42E-04
RBC	rs10495928	2	46206670	PRKCE	G/A	0.17	-0.09 ± 0.016	3.81×10^{-08}	[2]	-0.03 ± 0.007	1.11E-04
	rs3218097	6	42013253	USP49-MED20-BYSL-CCND3	T/C	0.19	0.10 ± 0.015	1.09×10^{-10}	[2]	0.04 ± 0.007	2.69E-10
	rs11966072	6	109741521	C6orf182-CD164	G/A	0.10	-0.11 ± 0.020	6.92×10^{-09}	[2]	-0.03 ± 0.009	1.98E-04
	rs7775698	6	135460328	HBS1L-MYB	T/C	0.35	-0.18 ± 0.012	7.31×10^{-48}	[2]	-0.07 ± 0.006	6.82E-34
	rs495828	9	135144688	ABO	T/G	0.28	-0.09 ± 0.013	3.33×10^{-12}	[2]	-0.03 ± 0.006	1.21E-06
	rs11611647	12	4204180	CCND2	C/T	0.37	-0.07 ± 0.012	6.44×10^{-09}	[2]	-0.03 ± 0.006	3.15E-07
WBC	rs3094212	6	31193749	CDSN-PSORS1C1	C/T	0.35	0.07 ± 0.012	6.76×10^{-09}	[2]	0.07 ± 0.029	1.25E-02
	rs4895441	6	135468266	HBS1L-MYB	G/A	0.37	-0.07 ± 0.012	1.67×10^{-09}	[2]	-0.12 ± 0.029	1.26E-05
	rs4065321	17	35397074	GSDMA-PSMD3-CSF3-MED24	T/C	0.32	-0.09 ± 0.012	2.94×10^{-14}	[2]	-0.16 ± 0.028	1.45E-08
PLT	rs5745568	6	33656372	BAK1	T/G	0.23	0.09 ± 0.014	6.66×10^{-11}	[2]	5.00 ± 1.163	1.72E-05
	rs7775698	6	135460328	HBS1L-MYB	T/C	0.34	0.09 ± 0.012	2.54×10^{-14}	[2]	5.28 ± 1.071	8.49E-07

rs739496	12	110372042	SH2B3	A/G	0.16	-0.14 ± 0.016	4.75 × 10^{-19}	[2]	-8.36 ± 1.572	1.06E-07

A1 is defined as minor allele and A2 is major allele. [1], Ganesh *et al.* 2009; [2], Kamatani *et al.* 2010;
